# ASL, DSC, DCE perfusion MRI and 18F-DOPA PET/CT in differentiating glioma recurrence from post-treatment changes

**DOI:** 10.1007/s11547-024-01862-3

**Published:** 2024-08-08

**Authors:** Giulia Moltoni, Andrea Romano, Gabriela Capriotti, Giuseppe Campagna, Anna Maria Ascolese, Allegra Romano, Francesco Dellepiane, Giuseppe Minniti, Alberto Signore, Alessandro Bozzao

**Affiliations:** 1 NESMOS, Department of Neuroradiology, S. Andrea Hospital, University Sapienza, Via di Grottarossa 1035/1039, 00189 Rome, Italy; 2 Department of Medical-Surgical Sciences and Translational Medicine, University of Rome “Sapienza”, Rome, Italy; 3 SMCMT Department, Radiotherapy Oncology, S. Andrea Hospital, University Sapienza, Rome, Italy; 4 Department of Radiological, Oncological and Pathological Sciences, “Sapienza” University of Rome, 00138 Rome, Italy; 5 IRCCS Neuromed, 86077 Pozzilli, Italy

**Keywords:** Glioma, Post-treatment changes, Tumor recurrence, PET, MRI perfusion

## Abstract

**Objectives:**

To discriminate between post-treatment changes and tumor recurrence in patients affected by glioma undergoing surgery and chemoradiation with a new enhancing lesion is challenging. We aimed to evaluate the role of ASL, DSC, DCE perfusion MRI, and 18F-DOPA PET/CT in distinguishing tumor recurrence from post-treatment changes in patients with glioma.

**Materials and methods:**

We prospectively enrolled patients with treated glioma (surgery plus chemoradiation) and a new enhancing lesion doubtful for recurrence or post-treatment changes. Each patient underwent a 1.5T MRI examination, including ASL, DSC, and DCE PWI, and an ^18^F-DOPA PET/CT examination. For each lesion, we measured ASL-derived CBF and normalized CBF, DSC-derived rCBV, DCE-derived Ktrans, Vp, Ve, Kep, and PET/CT-derived SUV maximum. Clinical and radiological follow-up determined the diagnosis of tumor recurrence or post-treatment changes.

**Results:**

We evaluated 29 lesions (5 low-grade gliomas and 24 high-grade gliomas); 14 were malignancies, and 15 were post-treatment changes.

CBF ASL, nCBF ASL, rCBV DSC, and PET SUVmax were associated with tumor recurrence from post-treatment changes in patients with glioma through an univariable logistic regression.

Whereas the multivariable logistic regression results showed only nCBF ASL (*p* = 0.008) was associated with tumor recurrence from post-treatment changes in patients with glioma with OR = 22.85, CI95%: (2.28–228.77).

**Conclusion:**

In our study, ASL was the best technique, among the other two MRI PWI and the 18F-DOPA PET/CT PET, in distinguishing disease recurrence from post-treatment changes in treated glioma.

## Introduction

Glioma is the most common primary malignant brain tumor in adults, with dismal overall survival for high-grade glioma and risk for recurrence and transformation into high-grade tumors for low-grade glioma. The current therapeutic protocol is based on surgery followed by chemotherapy and radiotherapy [1, 2].

The combined and adjuvant use of chemotherapy and radiation therapy improves survival but, on the other hand, increases the risk of developing post-treatment changes (PTC).

One of the most challenging differential diagnoses for neuroradiologists is distinguishing tumor recurrence (TR) from PTC. Indeed, even if these conditions are completely different in terms of pathophysiology [3, 4], they may appear similar on conventional MRI images showing edema and contrast enhancement [5]. To reach this goal, recent literature has focused on advanced imaging techniques [6].

Perfusion-weighted MRI (PWI) techniques have shown promising results [7], and to date, the most studied PWI sequence is dynamic susceptibility contrast (DSC)-enhanced perfusion [8–11]. DSC allows for the estimation of tissue microvascular density, especially through the measurement of cerebral blood volume (CBV) [12, 13], but it has issues such as the T1-relaxation effect, susceptibility artifacts, and the difficulty of evaluating small cortical lesions close to cortical vessels [14].

Dynamic contrast-enhanced (DCE) perfusion mainly investigates microvascular permeability [14]. Despite information from different DCE-derived parameters, it is still debated which of these parameters is most useful for the differential diagnosis between TR and PTC [15].

Compared to the other two PWI techniques, arterial spin labeling (ASL) perfusion has the advantage of not requiring the administration of contrast media, using arterial blood flow as an endogenous tracer, and allowing for tissue perfusion measurements through the measurement of cerebral blood flow (CBF) [14]. With the advent of the newer three-dimensional pseudocontinuous ASL (3D PCASL) [16, 17], this PWI may become a promising perfusion MRI technique in distinguishing recurrent neoplasm from treatment effects [18–27].

Parallel to advanced MRI sequences, PET examination with amino acid radiopharmaceutical may be helpful in differentiating TR from PTC [27], particularly 6-[^18^F]-fluoro-L-3,4-dihydroxyphenylalanine (^18^F-DOPA), which seems to be a valid tool to diagnose glioma recurrence [28, 29].

The purpose of our study is to evaluate the role of the three different PWI techniques (DSC, DCE, and ASL) and ^18^F-DOPA PET/CT in distinguishing TR from PTC in patients with glioma treated with standard-of-care surgery and chemoradiation. To our knowledge, this is the first comprehensive study of all three PWI methods and ^18^F-DOPA-PET/CT examination in a group of patients with glioma.

## Materials and methods

This study was approved by an institutional review board. Written informed consent was obtained from patients, and all clinical investigations were conducted according to the principles expressed in the Declaration of Helsinki.

### Study design and patients

From January 2021 to September 2023, 21 patients (11 men and 10 women; age range 28–76 years) who were being treated for glioma were prospectively and consecutively enrolled. Inclusion criteria were:

- Completion of standardized treatment, including gross total resection followed by radiation therapy and temozolomide chemotherapy.
- Presence of a contrast-enhanced lesion with findings inconclusive of TR or PTC on follow-up MRI performed at least 3 months after radiation treatment.
- No contraindications to undergo an MRI with contrast medium administration and an ^18^F-DOPA PET/CT examination.

The final diagnosis of TR or PTC was made based on radiologic and clinical evaluation with a final consensus decision made by a neuroradiologist (A.R. 15 years of experience) and a neurooncologist (A.M.A. 15 years of experience), both blinded to perfusion MRI and ^18^F-DOPA PET/CT data. Tumor recurrence was defined as a progressive increase in size, contrast enhancement, and mass effect despite steroid therapy in at least three subsequent MRI follow-up studies in an observational period of at least 9 months, in combination with deteriorating neurologic symptoms. Non-recurrence was defined on imaging as stable or resolving regions of enhancement over at least a 9-month observational period, accompanied by neurologic improvement during the follow-up period.

### Imaging acquisition

Each patient underwent an MRI and a PET/CT examination within 15 days of each other.

#### MRI

All MRI examinations were performed using a 1.5 T (Signa Voyager, GE) with a 32-channel array head coil. Along with conventional sequences (T1- and T2-weighted images, FLAIR, DWI with ADC map, and post-contrast 3D T1-weighted fast spoiled gradient-echo (FSPGR) images), the three PWI sequences were performed as follows:

- 3D PCASL before contrast-medium administration, labeling with a 3D stack-of-spirals fast spin echo readout (labeling duration: 1800 ms; post-labeling delay: 2025 ms; spiral interleaves: 8; points per spiral: 512; slice thickness: 4.0 mm; FOV: 24–26 cm; in-plane resolution: 3.64–4.53 mm^2^; bandwidth: 62.5 kHz; TE/TR:10.9/4840 ms; scan time: 4–5 min).
- DCE acquired before, during, and after the administration of a bolus of gadolinium-based MRI contrast agent (GBCA) followed by a saline flush using a 3D radial volumetric interpolated examination sequence (TR/TE: 4.5/1.6 ms; flip angle: 12°; slice thickness: 2.20 mm; FOV: 24–26 cm; bandwidth = 41.67 kHz; scan time: 8 min)
- DSC performed after DCE using the contrast medium agent previously administered for the DCE acquisition as a preload to correct the T1-relaxation effect. DSC was acquired before, during, and after the administration of a second bolus of GBCA followed by a saline flush using a single-shot gradient-echo echo planar imaging sequence (TR/TE, 1500/40 ms; flip angle: 60°; slice thickness: 4 mm; FOV 24–26 cm; bandwidth = 250 kHz; scan time: 90 s).

Gadoteric acid, with a total volume of 0.2 ml/kg of body weight and a flow of 3.5 ml/s, was used to accomplish both DCE and DSC, respectively, using 40% of the contrast volume for DCE acquisition and the remaining 60% for DSC scanning.

#### PET/CT

Patients underwent a protein fast for at least 4 hours. Patients did not receive pre-scanning Carbidopa administration. Twenty minutes after the injection of 2 MBq/kg of ^18^F-DOPA, a dedicated CT scan of the brain (120 kV, 80 mAs, 3 mm slice collimation) was performed, followed by a brain-centered static 3D PET (Biograph mCT, Siemens Healthcare, Erlangen, Germany) acquisition time of 15 min.

### Imaging analysis

#### MRI

Maps of ASL-derived CBF were generated from the 3D PCASL images using ReadyView ASL (GE Healthcare); maps of DSC-derived rCBV were generated from the DSC-MRI images using ReadyView BrainStat AIF (GE Healthcare); maps of DCE-derived transfer constant (Ktrans), fractional volume of the plasma space (Vp), fractional volume of the extravascular extracellular space (Ve), and reverse transfer rate constant (Kep) were generated from the DCE-MRI images using ReadyView GeniQ (GE Healthcare) with automatic computation of the Arterial Input Function. The ASL CBF, DSC rCBV, DCE Ktrans, DCE Vp, DCE Ve, and DCE Kep maps were co-registered with 3D post-contrast T1-weighted images using Volume Viewer (GE Healthcare), generating fused perfusion and contrast-enhanced images.

Using the co-registered images, distinct circular ROIs corresponding to the regions of contrast enhancement with the highest perfusion signal were put on each perfusion map by a board-certified neuroradiologist with 5 years of experience (G.M.), who was blinded to patient clinical information and PET/CT data. Susceptibility artifacts and vessels were avoided.

The normalized values (ASL nCBF) for the ASL perfusion were obtained by placing an extra ROI on the contralateral normal cerebral cortex. This ROI was created by dividing the mean signal intensity within the ROI at the region of contrast-enhancement by the mean signal intensity within the contralateral ROI.

#### PET/CT

Using scatter and attenuation correction, PET/CT images were rebuilt using the OSEM iterative technique (5 iterations, 24 subsets). Using Siemens SyngoVia software, ^18^F-DOPA PET was methodically merged to contrast-enhanced T1-weighted MRI sequences for visual reading. A qualified nuclear medicine physician (G.C., 15 years of experience) quantitatively examined the images. To calculate the maximum standardized uptake values (PET SUVmax), a spherical ROI was placed on the maximum lesion uptake.

The ROIs of the possible residual tumor were positioned at the location of the greatest MRI anomalies using a fused display of PET and post-contrast 3D T1-weighted imaging when no abnormal 18F-DOPA uptake was seen.

### Statistical analysis

Brunner Munzel test was performed to compare perfusion and ^18^F-DOPA PET/CT values in tumor recurrence and post-treatment changes. The symmetry/normality of these parameters was tested by Shapiro–Wilk test and checking of Q–Q plot.

A violin and box plots were performed to represent eventual differences between perfusion and ^18^F-DOPA PET/CT values in tumor recurrence and post-treatment changes.

Association between tumor recurrence from post-treatment changes in patients with glioma (dependent parameter) and ASL, DSC, DCE perfusion MRI, and ^18^F-DOPA PET/CT (independent parameters) was evaluated by univariable and multivariable logistic regression; in the latter the selection method was backward.

Univariable regression was performed to individually evaluate which independent parameters were associated with the dependent parameter.

Statistical analysis was performed using SAS version 9.4 TS Level 1 M8 and JMP PRO version 17 (SAS Institute, Cary, NC, USA).

A *p* value < 0.05 was considered statistically detectable.

## Results

Our inclusion criteria were met by 31 lesions (4 with low-grade gliomas and 17 with high-grade gliomas) in 21 patients. Two lesions (low-grade glioma) were excluded due to a change in patient treatment to a second-line option during follow-up. Out of the remaining 29 lesions that were part of the study, 15 turned out to be PTC and 14 turned out to be TR (5 low-grade gliomas and 24 high-grade gliomas) (see Table 1 for demographic characteristics).

**Table 1:** Demographic characteristics

Patient No.	Age (Years)	Sex	Lesion No.	Tumor type	WHO grade	Lesion site	RT dose (Gy)	Time from RT to imaging (months)	ChT	Lesion classification
1	63	F	1	GBM IDH-WT	IV	Right frontal	60	8	TMZ	PTC
2	71	M	2	GBM IDH-WT	IV	Left temporal	60	7	TMZ	TR
3	47	M	3	GBM IDH-MUT	IV	Right temporal	60	4	TMZ	TR
			4	GBM IDH-MUT	IV	Right temporal	60	4	TMZ	TR
4	58	M	5	Astrocytoma	II	Left parietal	60	7	TMZ	PTC
			6	Astrocytoma	II	Left parietal	60	7	TMZ	PTC
5	75	M	7	GBM IDH-WT	IV	Left parietal	40.5	8	TMZ	PTC
6	60	M	8	GBM IDH-WT	IV	Right temporal	37.5	6	TMZ	TR
			9	GBM IDH-WT	IV	Right parietal	37.5	6	TMZ	PTC
7	57	F	10	GBM IDH-WT	IV	Left temporo-parietal	60	12	TMZ	PTC
8	48	M	11	GBM IDH-WT	IV	Left frontal	60	6	TMZ	TR
			12	GBM IDH-WT	IV	Left frontal	60	6	TMZ	TR
9	60	F	13	GBM IDH-WT	IV	Left frontal	60	13	TMZ	PTC
			14	GBM IDH-WT	IV	Left frontal	60	13	TMZ	PTC
			15	GBM IDH-WT	IV	Callosal	60	20	TMZ	TR
10	60	M	16	GBM IDH-WT	IV	Right temporal	60	3	TMZ	TR
11	76	M	17	GBM IDH-WT	IV	Right parietal	60	10	TMZ	TR
12	28	F	18	Pleomorphic Xantho-astrocytoma	III	Left frontal	60	12	TMZ	PTC
13	54	M	19	Oligodendroglioma	II	Left frontal	50.4	24	TMZ	TR
			20	Oligodendroglioma	II	Left parietal	50.4	24	TMZ	TR
14	47	F	21	Oligodendroglioma	II	Right frontal	60	15	TMZ	PTC
15	34	M	22	Astrocytoma IDH-Mut	III	Right frontal	60	5	TMZ	TR
			23	Astrocytoma IDH-Mut	III	Right frontal	60	5	TMZ	TR
			24	Astrocytoma IDH-Mut	III	Right frontal	60	5	TMZ	PTC
16	60	F	25	GBM IDH-WT	IV	Left temporal	60	6	TMZ	PTC
17	32	F	26	Astrocytoma IDH-Mut	III	Left temporal	60	6	TMZ	PTC
18	52	F	27	Astrocytoma IDH-Mut	III	Left frontal	60	13	TMZ	PTC
19	56	F	28	Astrocytoma IDH-Mut	III	Left temporal	60	5	TMZ	TR
20	63	M	29	GBM IDH-WT	IV	Right parietal	60	7	TMZ	PTC

*RT*, radiation therapy; *ChT*, chemotherapy; *GBM*, glioblastoma, *IDH-WT*, isocitrate dehydrogenase-wild type; *IDH-Mut*, isocitrate dehydrogenase-mutant; *TMZ*, Temozolomide; *TR*, tumor recurrence; *PTC*, post-treatment changes

**Fig. 1:**
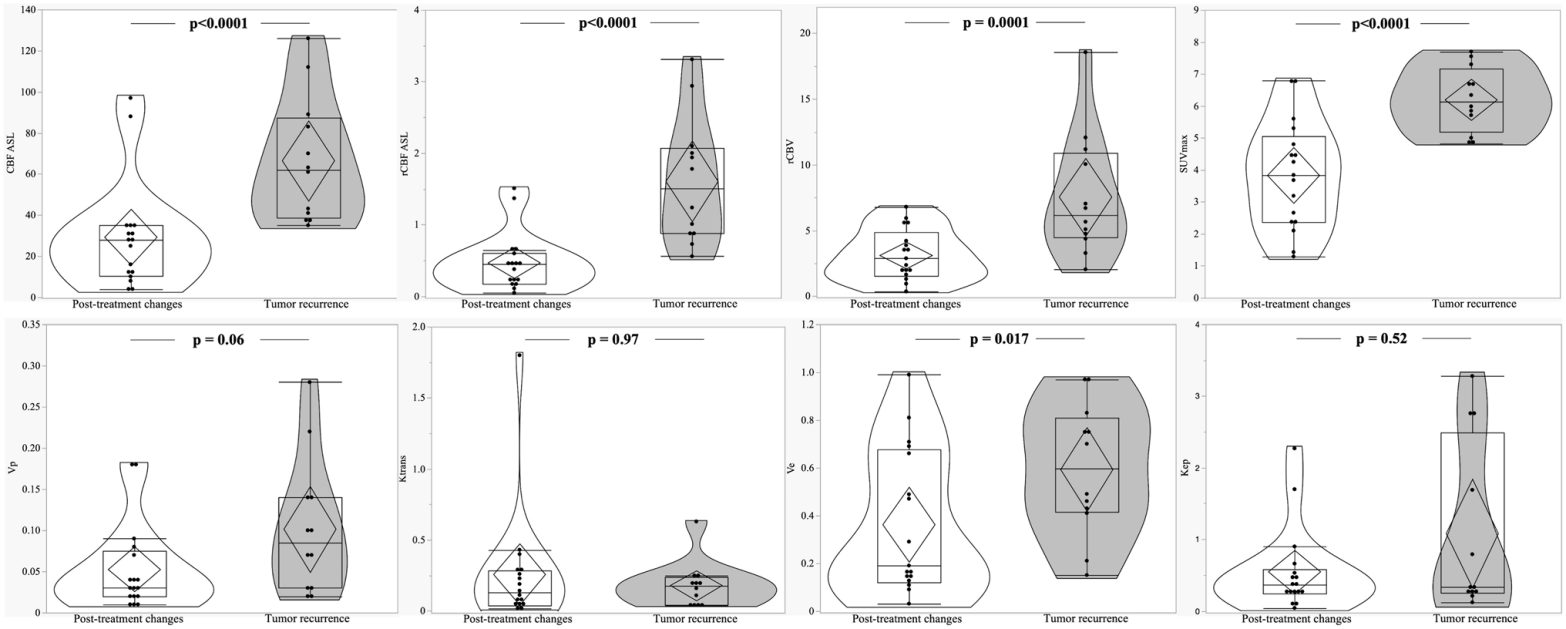
Box-plot comparing different parameters at post-treatment changes and tumor recurrence. **a** CBF_ASL, **b** nCBF_ASL, **c** rCBV_DSC, **d** SUVmax_PET; **e** Vp_DCE, **f** Ktrans_DCE, **g** Ve_DCE, **h** Kep_DCE). ASL = Arterial Spin Labeling; CBF = Cerebral Blood Flow; DSC = dynamic susceptibility contrast-enhanced perfusion; DCE = Dynamic contrast-enhanced perfusion; rCBV = relative cerebral blood volume; Kep = rate constant; Ktrans = transfer constant; Ve = fractional volume of the extravascular extracellular space; Vp = fractional volume of the plasma space; SUVmax = maximum standardized uptake values

We found detectable differences concerning ASL, DSC, DEC_Ve, and ^18^F-DOPA PET/CT comparing TR and PTC (Fig. 1). No similar results were obtained for the other DCE parameters.

**Fig. 2:**
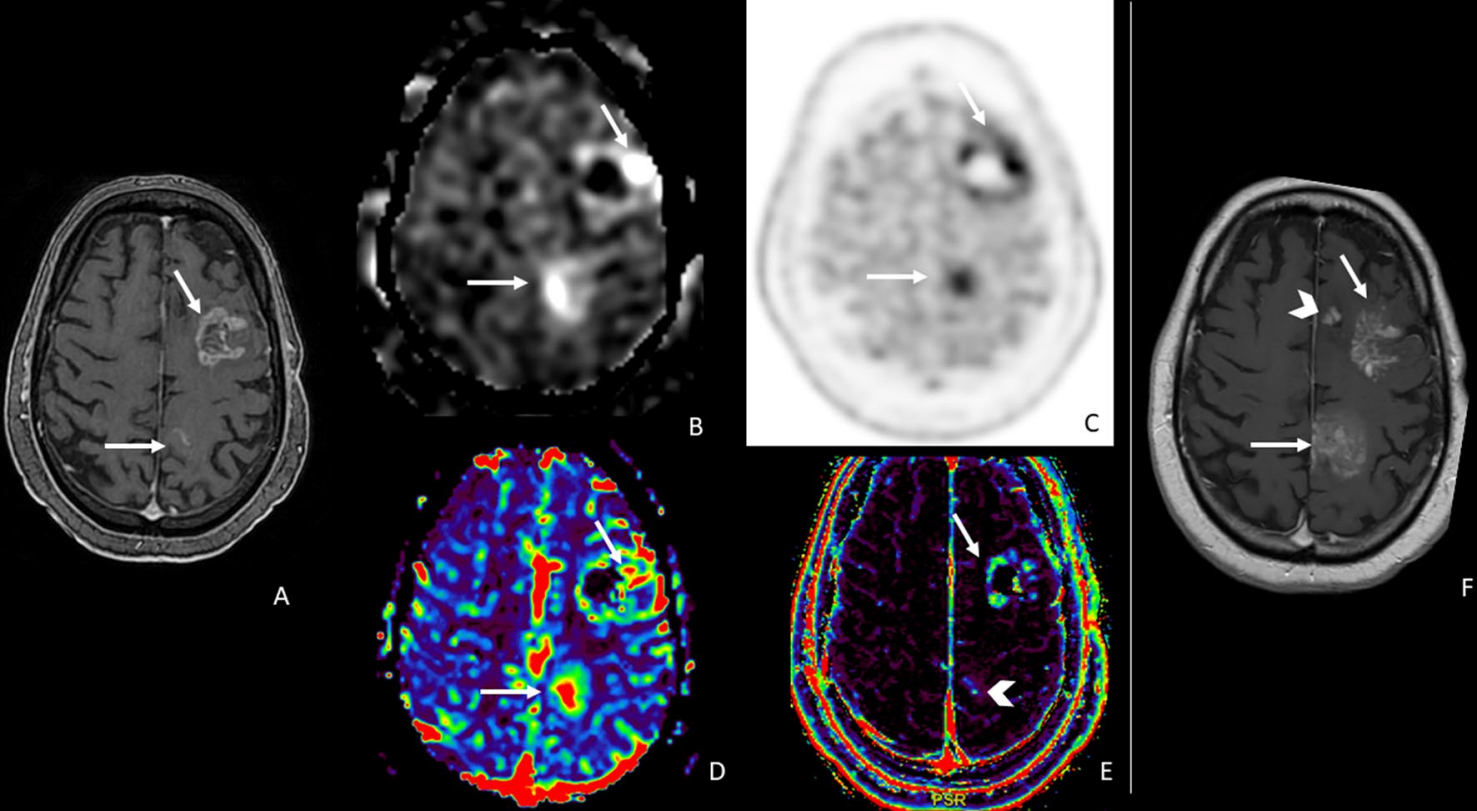
A representative case of high-grade glioma with two enhancing lesions in the left frontal lobe (**a**, arrows) appeared 6 months after radiation therapy. Lesions showed increased ASL_CBF (**b**, arrows), increased 18F-DOPA uptake (**c**, arrows), increased DSC_rCBV, particularly evident in the posterior lesion (**d**, arrows), doubtful DCE_Ve increase in the posterior lesion (**e**, arrowhead) and increased DCE_Ve values in the anterior lesion (f arrow). **f** 6-month follow-up MRI showing progression in lesion contrast enhancement and size, and a new lesion (arrowhead) as the expression of the disease recurrence. ASL, PET, and DSC correctly identify the tumoral nature of the lesions; Ve misclassified the posterior lesion. ASL = arterial spin labeling; CBF = cerebral blood flow; DSC = dynamic susceptibility contrast-enhanced perfusion; DCE = dynamic contrast-enhanced perfusion; CBV = cerebral blood volume; Ve = fractional volume of the extravascular extracellular space

**Fig. 3:**
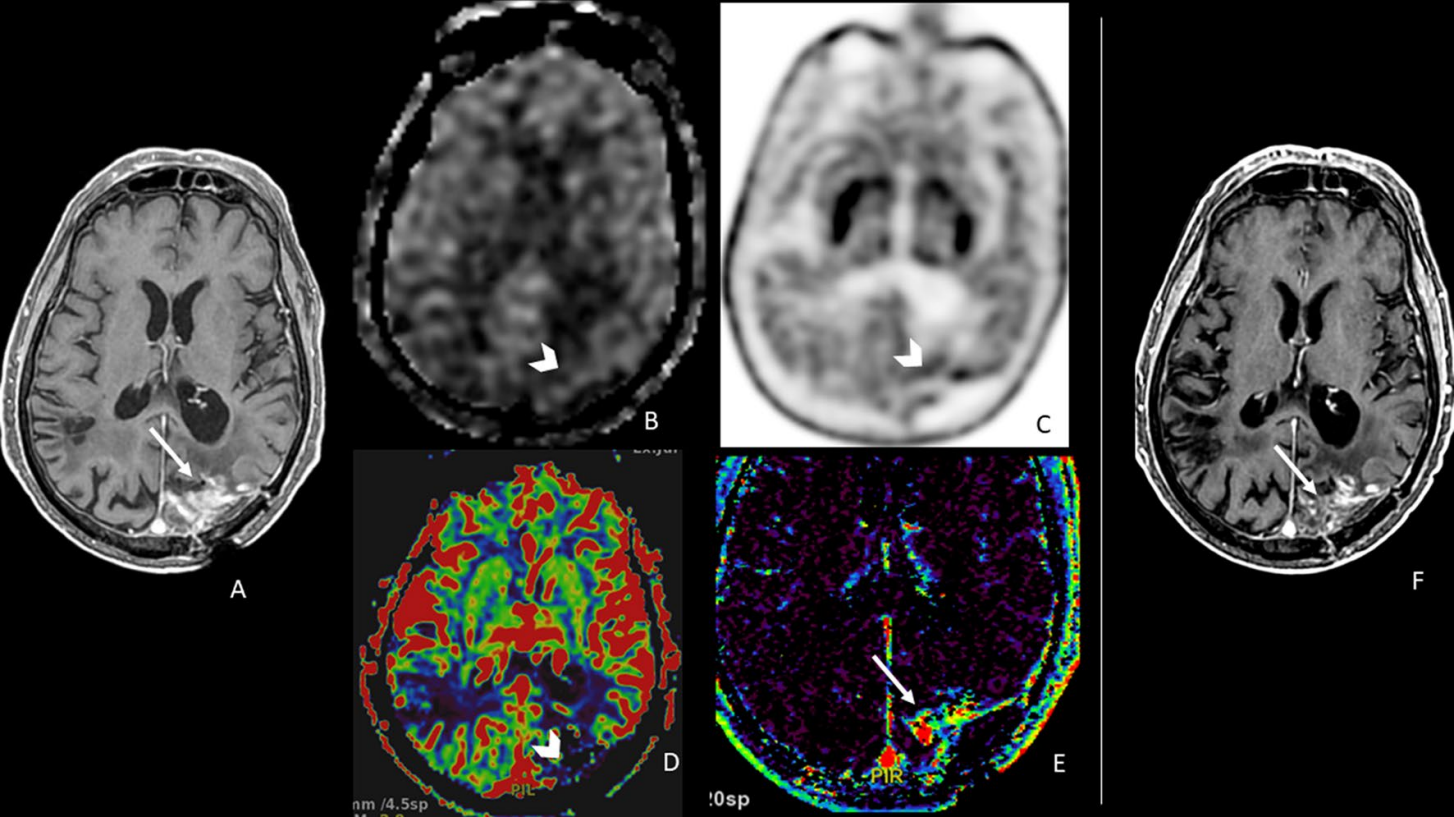
A representative case of a high-grade glioma with an enhancing lesion in the left parietal lobe (**a**, arrow) occurring 8 months after radiation therapy. The lesion did not show high values of ASL_CBF (**b**, arrowhead), DSC_rCBV (**d**, arrowhead), or a significant increase in 18F-DOPA PET uptake (**c**, arrowhead). DCE_Ve showed foci of increased values (**e**, arrow). Nine-month MRI follow-up showed the stability of the lesion with a slight reduction in contrast enhancement (**f**, arrow). The lesion was classified as post-treatment changes, and ASL, DSC, and PET were concordant and negative, whereas DCE_Ve misclassified the lesion. ASL = arterial spin labeling; CBF = cerebral blood flow; DSC = dynamic susceptibility contrast-enhanced perfusion; DCE = dynamic contrast-enhanced perfusion; CBV = cerebral blood volume; Ve = fractional volume of the extravascular extracellular space

Table 2, on the left side, showed univariable logistic regression results. Four parameters (CBF ASL, nCBF ASL, rCBV DSC, and PET SUVmax) were associated with tumor recurrence from post-treatment changes in patients with glioma (Figs. 2, 3)

**Table 2:** Univariable and multivariable logistic regression of tumor recurrence from post-treatment changes in patients with glioma according to MRI perfusion and PET parameters

Variable	Univariable logistic regression Odds Ratio (95%CI) (Beta ± SE)	*p*	Multivariable logistic regression Odds Ratio (95%CI) (Beta ± SE)	*p*
CBF ASL	1.05 (1.01 to 1.08) (0.05 ± 0.02)	**0.01**	0.41 (0.008 to 20.39) (− 0.88 ± 1.99)	0.66
nCBF ASL	22.85 (2.28 to 228.77) (3.13 ± 1.18)	**0.008**	22.85 (2.28 to 228.77) (3.13 ± 1.18)	**0.008**
Ktrans DCE	0.42 (0.02 to 7.28) (− 0.87 ± 1.46)	0.55	39.67 (− ∞ to + ∞) (3.69 ± 12.61)	0.77
Ve DCE	14.51 (0.95 to 222.59) (2.67 ± 1.39)	0.055	0.009 (− ∞ to + ∞) (− 4.69 ± 8.31)	0.57
Kep DCE	2.02 (0.81 to 5.06) (0.70 ± 0.47)	0.13	0.02 (− ∞ to 73.77) (− 4.01 ± 4.24)	0.34
Vp DCE	+ ∞ (0.22 to + ∞) (11.37 ± 6.57)	0.08	− ∞ (− ∞ to + ∞) (− 22.62 ± 25.84)	0.38
rCBV DSC	1.76 (1.11 to 2.78) (0.56 to 0.23)	**0.016**	17.45 (0.07 to + ∞) (2.86 to 2.82)	0.31
SUVmax	3.38 (1.39 to 8.24) (1.22 ± 0.45)	**0.007**	2.80 (0.08 to 103.36) (1.03 ± 1.84)	0.58

Statistically significant results are in bold (*p* < 0.05)

ASL = arterial spin labeling; CBF = cerebral blood flow; DSC = dynamic susceptibility contrast-enhanced perfusion; DCE = dynamic contrast-enhanced perfusion; rCBV = relative cerebral blood volume; Kep = rate constant; Ktrans = transfer constant; Ve = fractional volume of the extravascular extracellular space; Vp = fractional volume of the plasma space; SUVmax = maximum standardized uptake values

**Fig. 4:**
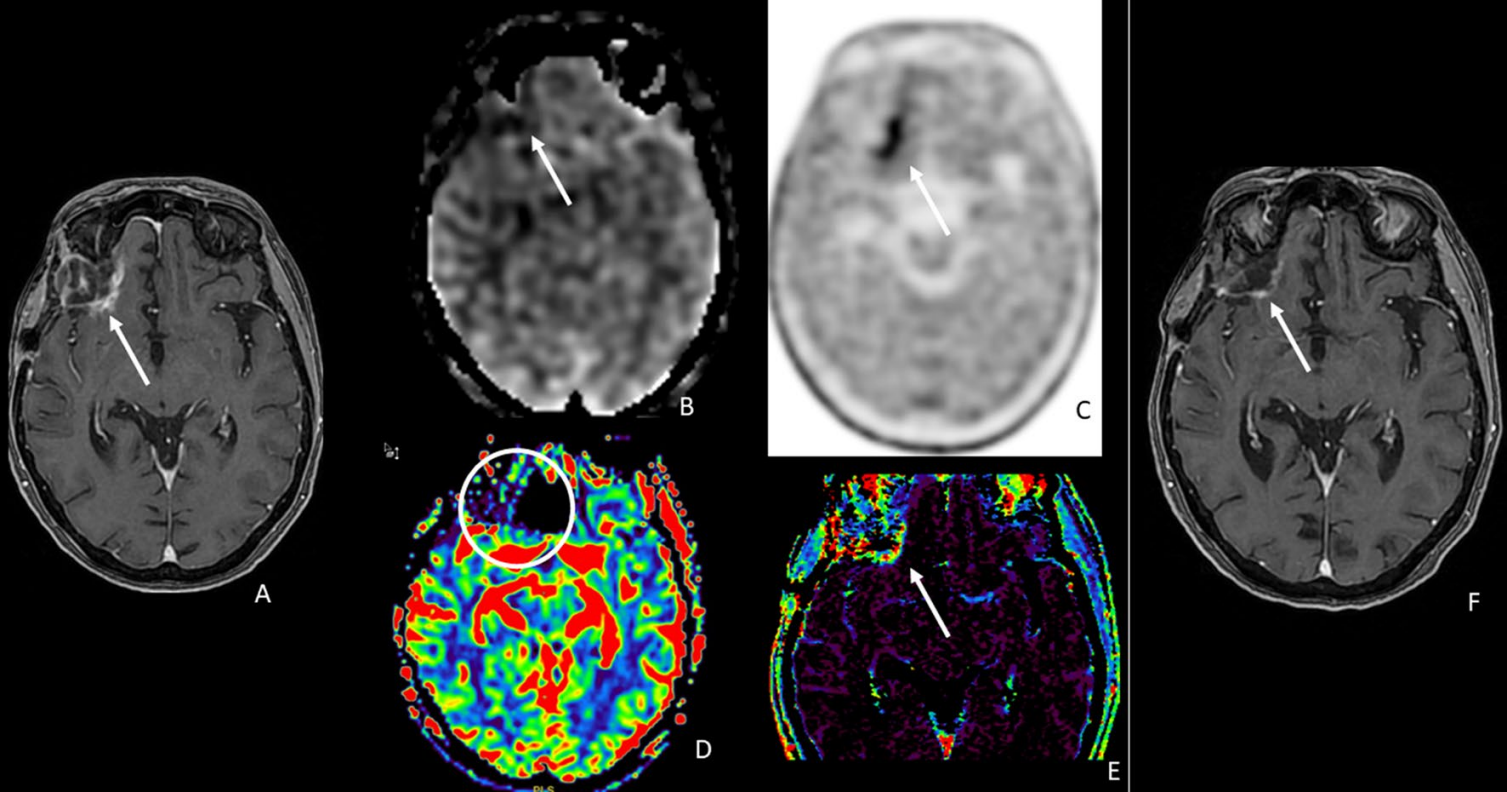
A representative case of high-grade glioma with a linear enhancing lesion in the left front-basal region, close to the surgical site, occurred 8 months after radiation therapy (**a**, arrow). The enhancing lesion did not show an increase in ASL_CBF (**b**, arrow); DSC-rCBV evaluation was difficult because of the proximity to bone structures (**d**, circle); and DCE_Ve was slightly increased (**e**, arrow). The uptake of 18F-DOPA was high (**c**, arrow). Six months of MRI follow-up showed a massive reduction in contrast enhancement (**f**, arrow). ASL correctly classified the lesion, whereas the linear 18F-DOPA uptake close to the surgical site could be the expression of macrophages activation. ASL = arterial spin labeling; CBF = cerebral blood flow; DSC = dynamic susceptibility contrast enhanced perfusion; DCE = dynamic contrast enhanced perfusion; CBV = cerebral blood volume; Ve = fractional volume of the extravascular extracellular space

**Fig. 5:**
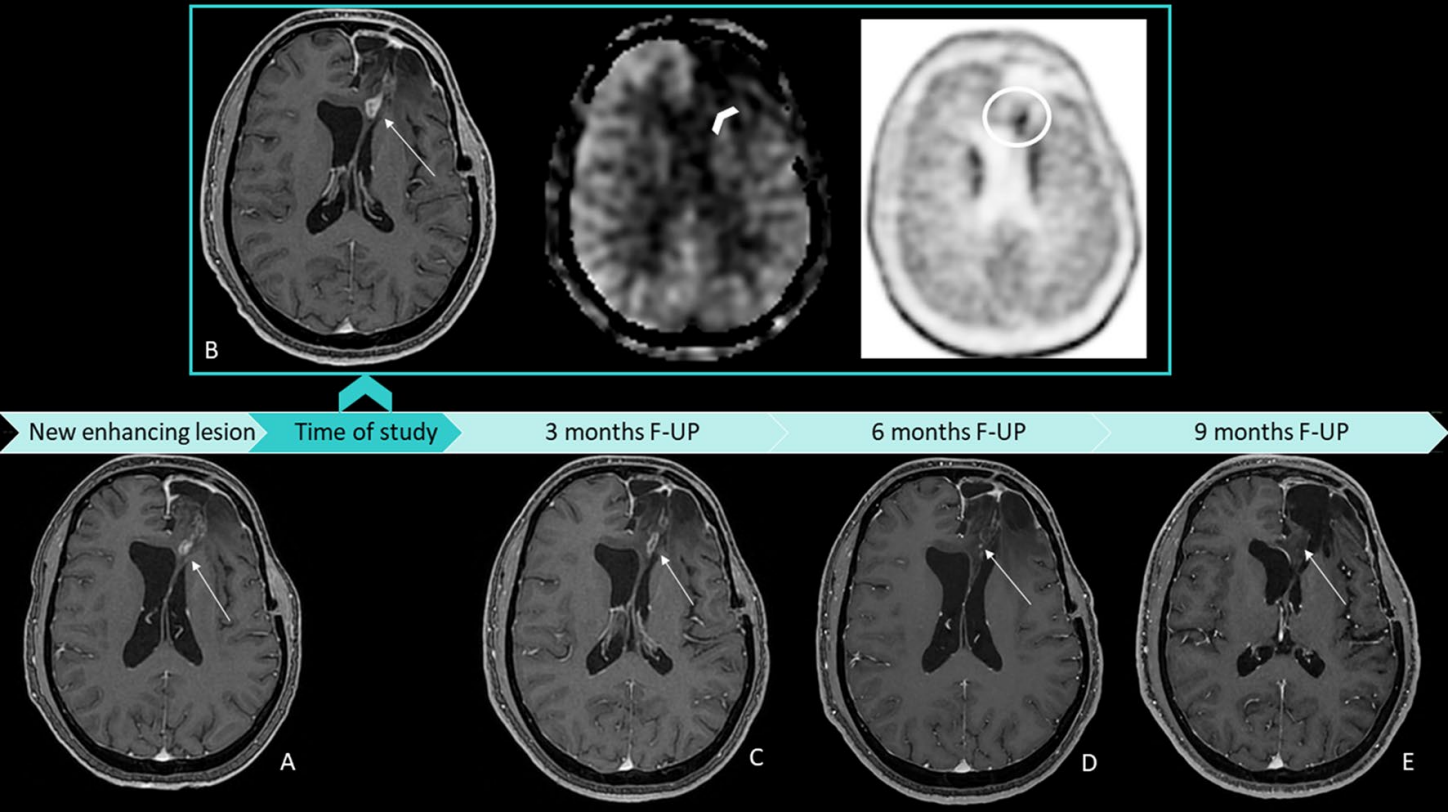
A representative case of high-grade glioma with a new enhancing lesion appeared 3 months after radiation therapy (**a**, arrow); the patient was included in our study, and after 2 months he underwent MRI and PET examination following study protocol (**b**, squared images). On T1 post-contrast WI, the lesion was increased in size (**b**, arrow), the ASL_CBF map showed no increment of CBF (**b**, arrowhead), and 18F-DOPA PET/TC showed an increased uptake (**b**, circle). T1 post-contrast WI acquired, respectively, after 3 (**c**) and 6 (**d**) months, showed a progressive reduction of contrast enhancement and lesion size until, after 9 months (**e**), the lesion was no longer present. This is a case of pseudoprogression with a false positive PET/CT and a true negative ASL classification. ASL = arterial spin labeling; CBF = cerebral blood flow; DSC = dynamic susceptibility contrast-enhanced perfusion; DCE = dynamic contrast-enhanced perfusion; CBV = cerebral blood volume; Ve = fractional volume of the extravascular extracellular space; WI = weighted imaging

Table 2, on the right side, reported the results of the multivariable logistic regression. Only nCBF ASL (*p *= 0.008) was associated with tumor recurrence from post-treatment changes in patients with glioma with OR = 22.85, CI95%: (2.28–228.77) (Figs. 4, 5). Particularly, patients who showed an increase in this parameter had a risk of recurrence of approximately 23 times compared to subjects who did not experience it.

## Discussion

To differentiate disease recurrence from PTC in the follow-up MRI examination of patients with treated glioma is challenging and, at the same time, crucial in terms of patient management and prognosis.

In order to distinguish PTC from glioma recurrence, we assessed the diagnostic predictivity of ASL-PWI, DSC-PWI, DCE-PWI, and ^18^F-DOPA PET/CT in this investigation.

To date, only Seeger et al. [30] and Nguyen et al. [24] have compared all three PWI sequences; nobody all three PWI methods and ^18^F-DOPA-PET/CT in a group of patients with glioma.

Our results have highlighted that when the diagnostic techniques were individually assessed through a univariable analysis, three of them showed association in assessing recurrence compared to post-treatment changes in glioma: ASL-PWI (both the absolute and the normalized CBF), DSC-PWI, and ^18^F-DOPA PET/CT. On the other hand DCE-PWI did not show statistically detectable results.

Anyway, when the examined parameters were assessed through a multivariate analysis, the only statistically detectable parameter was the nCBF ASL, with a 23 times higher risk of disease recurrence in case of elevated nCBF values.

ASL superior performance to the other two PWI sequences is in concordance with the most recent literature.

DSC-PWI seems to be a quite useful technique for differentiating between glioma recurrence and radio-induced sequelae, as demonstrated by our univariable analysis, and has largely been described in the literature [16, 31]. However, when compared with PCASL, DSC-PWI loses its diagnostic power probably due to some issues intrinsic to the sequence such as the susceptibility artifacts when blood products are present, and the difficulty of identification of small or close to cortical vessels lesions. Manning et al. affirmed that ASL-nCBF has a higher AUC than DSC-derived parameters in differentiating progressive disease from pseudo-progression in treated glioblastoma [32], Wang et al. confirmed that 3D PCASL is a suitable substitute for DSC in separating radiation-induced brain injury from glioma recurrence [19], and Xu et al. found that PCASL is superior to DSC in discriminating between PTC and glioma recurrences [20]. Whereas Seeger et al. reported poor ASL performance [30]; anyway, this could be related to the use of a pulsed ASL (PASL) rather than the newest and recommended 3D PCASL [18, 33].

Concerning DCE-PWI, our results showed that only Ve was statistically significant when referring to box-plot graphics. Anyway in univariate and multivariate analysis none of DCE-derived parameters were useful in the predictivity of TR from PTC in treated glioma. In the literature, DCE results are not robust on this topic [34], with some authors reporting the best performance for Ktrans [35, 36], others for Ve [37], and others affirming the inferiority of DCE to DSC [3, 38]. We can speculate that the poor performance of DCE can be explained by the presence of permeability damages both in recurrences and in PTC.

Conversely, an increased CBF in glioma recurrences is easily explained by the abundant neoangiogenesis occurring in recurrences to supply the metabolic demand of tumoral tissue, whereas in radiation-induced brain injury, there is vascular dilation and endothelial injury without an increase in vascular density and consequently without an increased CBF. One of the most known ASL-PWI limits concerns inter-subject and intra-subject variability of CBF measurements, which could be influenced by multiple variables some patient-related (i.e., patient age, patient atherosclerotic condition) and others depending on sequence setting (i.e., labeling duration, post-labeling delay). Normalizing the CBF value for the contralateral cortex may help mitigate all these variables, and we believe that this is the reason why in the multivariate analysis the only statistically detectable parameter is the normalized CBF and not the absolute CBF value.

Concerning ^18^F-DOPA PET/CT, in concordance with previous research [29], our study reported good performance for PET SUVmax in univariate analysis and plot graphics. In the literature, it has been described the efficacy of ^18^F-DOPA PET for diagnosis, prognosis, and treatment evaluation of patients with low and high-grade glioma, especially when compared with not advanced MRI sequences [39]. Particularly, ^18^F-DOPA could be extremely useful when MRI findings are negative or inconclusive in naive or recurrent neoplastic lesions [39].

Anyway, ^18^F-DOPA PET/CT is not statistically detectable when compared to the other techniques in multivariate analysis. We can speculate that this is imputable to some limits of this technique. Some authors reported cases of false positive uptake along surgical margins, caused by post-operative inflammatory changes and expression of amino acid transporter in activated macrophages [40, 41]

More recently, Chiaravalloti et al. [42] found that a high ^18^F-DOPA uptake soon after radiotherapy may be treatment-related and advised to be careful in this cohort of patients. A more recent systematic review [43] confirmed these limits, affirming that ^18^F-DOPA PET specificity for detecting recurrent gliomas was not optimal due to increased false positive rates caused by treatment responses such as edema and inflammatory tissue. Finally, Pellerin et al. [44] showed that ^18^F-DOPA PET is more inclined to misdiagnose pseudo-progression than ASL, reporting 5 false positives out of 58 cases by analyzing SUVmax compared to zero false positives based on ASL CBF [44]. In concordance with those studies, we found a few ^18^F-DOPA PET/CT false positive cases in our sample related to an uptake along surgical margins (Fig. 4), and to pseudo-progression (Fig. 5).

Berteaux et al. analyzed the role of the hybrid PET/MRI scanners using ^18^F-DOPA in treated glioma [45], showing how ASL sequences may help mitigate the lower specificity of ^18^F-DOPA PET in hemorrhagic lesions with macrophage activation with an AUC curve of 0.93 in the combined ASL/PET analysis, which is very similar to our ASL nCBF AUC curve (0.926) and superior to our PET SUVmax AUC curve (0.882) [45].

So, the hybrid PET/MRI scanners using ^18^F-DOPA seem to be not so superior to ASL MRI alone, in comparison with the great advantage of using a noninvasive MRI perfusion without the use of exogenous tracers, with possible positive repercussions on patient safety and health costs. For the same reasons, ASL MRI in the routine follow-up of patients with treated gliomas may avoid additional PET/CT examinations, sparing in terms of patient ionizing radiation exposure and health costs.

Our study has several limitations. First, the small and heterogeneous sample of patients, including high-grade and low-grade gliomas, could have affected the performance we reported. Thus, a study with a large and homogeneous sample is needed to validate the imaging techniques on the object. Second, a pathology examination was not available for confirmation of the diagnosis. Third, the use of a 1.5T MRI scanner rather than a 3T could have had an impact on ASL and DCE evaluation. Particularly, we cannot exclude that the poor DCE performance in our study could be related to the 1.5T scanner. Anyway, a recent meta-analysis on DCE, including studies performed at a 3T scanner, confirmed the moderate diagnostic accuracy of DCE in discriminating recurrent lesions from treatment-related changes in glioma [46]. Concerning ASL, no significant differences have been reported in the emerging literature when comparing PCASL acquired at 1.5T versus 3T [47, 48]. Indeed, Baas et al. affirmed that ASL imaging is reproducible at both field strengths, and even if scanning at 3T has the advantage of having a higher signal-to-noise ratio and allowing more extensive acquisition parameter optimization, ASL is a cost-effective and safe alternative to contrast agent-based perfusion at both field strengths [47]. Moreover, our ASL results, with particular regard to normalized CBF, are robust and in agreement with the literature suggesting that even a PCASL acquired at 1.5 T is a valid and helpful tool in discriminating glioma recurrence from radiation-induced changes.

## Conclusion

With higher values in tumor recurrences as the expression of neoangiogenesis, PCASL seems to be the imaging modality that best predicts TC from PTC among the three MRI-PWI sequences and ^18^F-DOPA PET/CT.

nCBF ASL-derived parameter resulted to be the best parameter correlated to tumor recurrence.

DSC and 18F-DOPA PET/CT are valid tools with some limits such as susceptibility artifacts, difficult evaluation of cortical lesions for DSC, and possible false positives in case of pseudo-progression or uptake alongside surgical margins for ^18^F-DOPA PET/CT. DCE does not reach a good diagnostic performance.

In summary, ASL outperformed the other methods under investigation, and it also had the advantages of being simple to acquire and not requiring contrast medium injection.

## Data Availability

Data generated or analyzed during the study are available from the corresponding author by request.

## Abbreviations

ASL: Arterial spin labeling
CBF: Cerebral blood flow
nCBF: Normalized cerebral blood flow
rCBV: Relative cerebral blood volume
DCE: Dynamic contrast-enhanced perfusion
DSC: Dynamic susceptibility contrast-enhanced perfusion
Kep: Rate constant
Ktrans: Transfer constant
PTC: Post-treatment changes
SUV_max: Maximum standardized uptake values
TR: Tumor recurrence
Ve: Fractional volume of the extravascular extracellular space
Vp: Fractional volume of the plasma space18F-DOPA=6-[18F]-fluoro-L-3,4-dihydroxyphenylalanine
